# Successful Endovascular Entry Closure for Retrograde Type A Aortic Dissection Originating from the Right Subclavian Artery: A Case Report

**DOI:** 10.3400/avd.cr.25-00056

**Published:** 2025-07-17

**Authors:** Haruo Suzuki, Shoji Sakaguchi, Bunpachi Kakii, Gaku Uchino, Masato Furui, Norikazu Oshiro, Shinichi Mitsuyama, Yasutaka Hirai, Takeshi Yoshida

**Affiliations:** 1 Department of Cardiovascular Surgery, Matsubara Tokushukai Hospital, Matsubara, Osaka, Japan; 2 Department of Radiology, Matsubara Tokushukai Hospital, Matsubara, Osaka, Japan; 3 Department of Cardiovascular Surgery, Nishinomiya Watanabe Cardiovascular Cerebral Center, Nishinomiya, Hyogo, Japan

**Keywords:** coronary angiography, type A aortic dissection, entry closure

## Abstract

Iatrogenic type A aortic dissection (TAAD) is a rare but potentially fatal complication of coronary angiography. We report a case of iatrogenic retrograde TAAD originating from the right subclavian artery. Endovascular entry closure using a stent graft led to resolution of the false lumen and favorable aortic remodeling. The patient remained free from cardiovascular events over a 4-year follow-up. This case highlights the potential efficacy of endovascular treatment, even in retrograde TAAD with its entry located in the subclavian artery.

## Introduction

Iatrogenic type A aortic dissection (TAAD) is a rare but life-threatening complication that can occur during coronary angiography (CAG) or percutaneous coronary intervention (PCI). Although surgical repair remains the standard treatment for TAAD, the feasibility of endovascular treatment in cases with retrograde extension from peripheral arteries, such as the subclavian artery, remains unclear because of limited clinical data. Here, we report a case of iatrogenic retrograde TAAD with an entry tear at the right subclavian artery, which was successfully managed by endovascular entry closure using a stent graft, resulting in a favorable mid- to long-term outcome.

## Case Report

**Figure 1:**
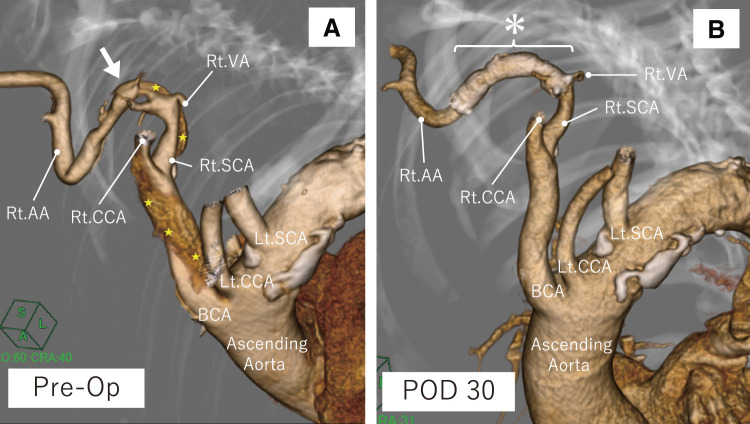
Fig. 1 Three-dimensional reconstructions of computed tomographic angiography before and after the procedure. (**A**) A markedly tortuous Rt. SCA is observed, with the entry tear identified distal to the Rt. SCA. (**B**) The Rt. VA remains patent, and the stent graft is positioned appropriately. Arrow: entry; Star: false lumen; Asterisk: stent graft. Rt.: right; Lt.: left; BCA: brachiocephalic artery; CCA: common carotid artery; SCA: subclavian artery; AA: axillary artery; VA: vertebral artery; Op: operation; POD: postoperative day

A 77-year-old woman with a medical history of dyslipidemia and mild hypertension underwent CAG via the right radial artery for routine follow-up evaluation 1 year after PCI for unstable angina. The operator encountered significant tortuosity in the right subclavian artery, which made catheter advancement challenging (**Fig. 1A**). Although the catheter reached the sinus of Valsalva, it failed to engage the coronary ostia. A test injection demonstrated radiographic findings consistent with an aortic dissection involving the ascending aorta. The patient remained asymptomatic and hemodynamically stable. Consequently, vascular access was changed to the right common femoral artery, and diagnostic CAG was completed. No coronary artery dissection or significant stenosis was observed, and there was no evidence of coronary ostial compromise due to false lumen compression. Subsequent non-contrast and contrast-enhanced computed tomography (CT) confirmed the diagnosis of TAAD, and the patient was referred to our department for further management.

**Figure 2:**
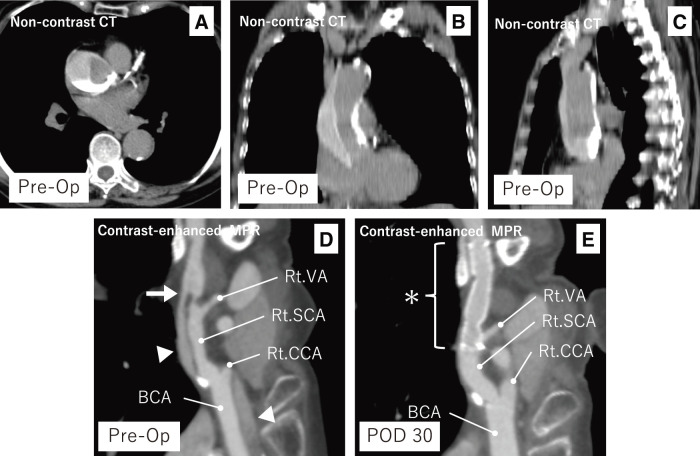
Fig. 2 Preoperative non-contrast CT (axial, coronal, and sagittal; **A**–**C**) and contrast-enhanced curved MPR images (preoperative day and POD 30; **D**, **E**). (**A**–**C**) Contrast pooling is visible along the posterior wall of the ascending aorta. (**D**) A focal intimal defect is seen in the distal right subclavian artery, with strong contrast enhancement at this level and gradual attenuation proximally. (**E**) The stent graft covers the entry site, and the false lumen has resolved. Arrow: entry; Arrowhead: false lumen; Asterisk: stent graft. VA: vertebral artery; SCA: subclavian artery; CCA: common carotid artery; BCA: brachiocephalic artery; MPR: multiplanar reconstruction; Op: operation; POD: postoperative day

**Figure 3:**
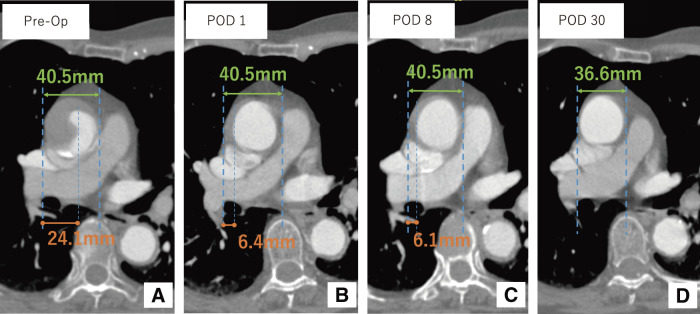
Fig. 3 Axial contrast-enhanced CT images obtained preoperatively (single image) and postoperatively (multiple images). (**A**) Pre-treatment image shows true lumen compression and contrast pooling in the false lumen. The superior vena cava is compressed by the enlarged aorta. (**B**) On POD 1, the true lumen is expanded and the false lumen thickness is reduced. (**C**) The false lumen thickness continues to decrease. (**D**) By POD 30, the false lumen is no longer visible and the aortic diameter has decreased. Op: operation; POD: postoperative day

Non-contrast CT performed after CAG revealed contrast pooling forming a fluid level from the dorsal to the lesser curvature side of the ascending aorta (**Figs. 2A**–**2C**). This finding was interpreted as residual contrast medium from the CAG procedure. On subsequent contrast-enhanced CT, after accounting for the pooled contrast, strong contrast enhancement was observed in the distal right subclavian artery, with gradual attenuation toward the aortic root. A focal intimal defect was identified at the site of maximal enhancement, consistent with an entry tear (**Fig. 2D**). Based on the enhancement pattern and tear location, we diagnosed retrograde TAAD originating from the right subclavian artery. The false lumen extended proximally to the sinus of Valsalva and terminated distally as a blind end within the aortic arch. No re-entry sites were identified. The maximal diameter of the ascending aorta was 40.5 mm, with a false lumen thickness of 24.1 mm (**Fig. 3A**).

Given the absence of symptoms, stable hemodynamics, and localized contrast pooling within the false lumen, it was determined that closure of the entry site via the subclavian artery would be an appropriate definitive treatment. Surgical intervention was considered; however, due to the patient’s advanced age and comorbidities, it was deemed excessively invasive. Endovascular repair with a stent graft was selected as a less invasive and anatomically feasible alternative.

In this case, contrast-enhanced CT strongly suggested that the entry was located in the right subclavian artery near the origin of the right vertebral artery. Considering the potential risk of vertebral artery occlusion during endovascular treatment, evaluation of the patency of the circle of Willis was deemed important for selecting an appropriate therapeutic approach. Although we were aware of the potential for sudden hemodynamic deterioration, the patient’s condition had remained consistently stable since onset. Therefore, magnetic resonance angiography (MRA) was performed based on a multidisciplinary discussion among cardiologists, cardiovascular surgeons, and radiologists, and the clinical benefit of obtaining cerebral circulation information was considered to outweigh the risk. Preoperative MRA confirmed adequate collateral circulation between the bilateral vertebral arteries, thereby mitigating the potential risk of right vertebral artery occlusion during and after stent graft deployment.

After completing diagnostic imaging and risk assessment, endovascular treatment was urgently performed on the same day as the CAG. The procedure was performed under local anesthesia and mild sedation. A 4-Fr sheath introducer (Medikit, Tokyo, Japan) was inserted into the right brachial artery, and an 8-Fr sheath introducer (Medikit) was placed in the left common femoral artery. Initial attempts to retrogradely pass a 0.035-inch guidewire (Radifocus; Terumo, Tokyo, Japan) through the true lumen of the dissected right subclavian artery from the brachial approach were unsuccessful. Therefore, an 8-Fr sheath was used via the left femoral artery to advance a 0.035-inch guidewire and a 4-Fr JB2-type diagnostic catheter (CX catheter; Gadelius Medical, Tokyo, Japan) into the brachiocephalic artery. The true lumen of the right subclavian artery was successfully accessed in an antegrade fashion. A pull-through system was established by retrieving the guidewire through the right brachial sheath. The guidewire was then exchanged for a 0.014-inch stiff-type wire (Aguru; Boston Scientific, Marlborough, MA, USA). Intravascular ultrasound (IVUS) was utilized to confirm the true lumen placement of the guidewire and identify the approximate location of the entry tear. While IVUS was used as a supplementary reference, final device sizing was determined based on a combination of imaging findings. An 8 × 50-mm stent graft (Viabahn; W.L. Gore & Associates, Newark, DE, USA) was introduced through the femoral artery using the pull-through technique and deployed with care to avoid occlusion of the right vertebral artery. Post-deployment angiography confirmed complete sealing of the entry tear, with no residual flow into the false lumen.

The postoperative course was uneventful. The patient remained hemodynamically stable, and there were no signs of cardiac tamponade, organ malperfusion, or neurological deficit. CT on postoperative day 1 demonstrated that the false lumen appeared largely thrombosed. Compared to preoperative imaging, the false lumen thickness was reduced to 6.4 mm, likely reflecting decreased flow and early thrombus organization, while the ascending aortic diameter remained unchanged (**Fig. 3B**). Notably, the dorsal contrast pooling that had been visible in the ascending aorta on preoperative imaging was no longer observed, supporting the interpretation of substantial thrombus formation. By postoperative day 8, the false lumen thickness was further reduced to 6.1 mm (**Fig. 3C**). The patient was discharged on postoperative day 10 with independent ambulation. The decision to discharge was based on her stable clinical status, decreasing levels of C-reactive protein and D-dimer, and consistent blood pressure control during early rehabilitation. Close follow-up was planned, considering the potential for morphological changes in the acute phase of TAAD. At follow-up CT on postoperative day 30, the ascending aortic diameter had slightly decreased to 36.6 mm, and the false lumen had almost resolved, indicating favorable aortic remodeling (**Figs. 1B**, **2E**, and **3D**). At 4 years postoperatively, the patient remains asymptomatic with no recurrence of dissection or cardiovascular events, and no evidence of stent graft migration, stenosis, or occlusion.

## Discussion

Iatrogenic TAAD is a rare but potentially fatal complication of diagnostic and interventional coronary procedures. The reported incidence ranges from 0.02% to 0.07% and is significantly lower during diagnostic procedures (0.01%) than during interventional ones (0.04%).^1–4)^ Dissection is typically induced by mechanical trauma to the arterial wall, often during manipulation of guidewires or catheters, especially in tortuous or calcified vessels. In coronary arteries, additional risks include balloon inflation or stent deployment.

In the present case, retrograde TAAD occurred during CAG via a right radial approach, due to catheter manipulation in a severely tortuous right subclavian artery. Despite retrograde extension into the ascending aorta and antegrade propagation into the aortic arch, the patient remained asymptomatic and hemodynamically stable. Timely endovascular sealing of the entry tear in the subclavian artery led to thrombus formation within the false lumen, followed by gradual thrombus resorption. By postoperative day 30, the false lumen was no longer visible on CT, indicating favorable aortic remodeling. The patient remained free from cardiovascular events over a 4-year follow-up period.

Several previously reported cases provide insight into the clinical variability and diversity of treatment approaches for iatrogenic retrograde TAAD. Tochii et al. described a stable patient with retrograde TAAD originating from the left subclavian artery who underwent ascending aortic replacement prophylactically due to concern for future rupture.^5)^ Conversely, Kim et al. reported a case involving cardiac tamponade and hemodynamic instability, which was ultimately managed conservatively, with stabilization occurring prior to the initiation of open surgery.^6)^ Khan et al. and Manan et al. also described cases with subclavian or brachiocephalic artery entries that were successfully treated without surgical intervention in clinically stable patients.^7,8)^

These reports demonstrate a spectrum of therapeutic strategies, ranging from prophylactic surgical intervention to conservative management. In the absence of established treatment guidelines or consensus-based protocols for retrograde TAAD, therapeutic decisions have relied largely on individual clinical judgment. Although endovascular repair has not yet been widely reported for retrograde TAAD cases originating from peripheral vessels, our case provides preliminary evidence suggesting its technical feasibility and potential for favorable anatomical outcomes in selected patients.

The selection of stent graft size in arterial dissection is a critical factor that directly impacts treatment outcomes. However, there is no established consensus on the optimal sizing strategy, and decisions vary among operators and institutions. Excessive oversizing may increase the risk of new entry formation, whereas undersizing can result in inadequate sealing and lead to endoleaks. At our institution, we aim to estimate the native arterial diameter prior to dissection using CT or IVUS and to select a stent graft with a size close to that diameter or with no more than approximately 10% oversizing. In this case, severe stenosis at the proximal landing zone precluded accurate estimation of the normal diameter. Therefore, we referred to the distal true lumen diameter assessed by IVUS and corroborated this with the contralateral left subclavian artery diameter measured on preoperative CT, leading to the selection of an 8 × 50-mm stent graft.

Our outcome aligns with findings from type B aortic dissection studies. The INSTEAD-XL trial by Nienaber et al. demonstrated that patients in whom false lumen flow was occluded showed significantly greater aortic remodeling and improved long-term outcomes compared to those managed with optimal medical therapy alone.^9)^ Furthermore, Kinoshita et al. reported that earlier occlusion of false lumen flow resulted in better aortic remodeling than delayed intervention.^10)^ In the present case, early entry closure also led to favorable aortic remodeling and stable mid- to long-term outcomes. Based on these findings, we hypothesize that, despite anatomical differences, early occlusion of false lumen flow in arterial dissection may universally contribute to improved arterial remodeling and prognosis.

This case emphasizes the potential of endovascular entry closure as a less invasive and effective alternative to open surgery for retrograde TAAD originating from peripheral vessels. In anatomically and hemodynamically suitable patients, this approach may represent a valuable addition to the therapeutic armamentarium.

## Conclusion

This case illustrates that even retrograde TAAD with a peripheral entry can be successfully treated with a less invasive endovascular approach. In anatomically suitable and hemodynamically stable patients, entry closure may offer a favorable prognosis and serve as a viable alternative to open surgery. Further studies are warranted to establish appropriate indications and validate its broader applicability.

## Declarations

### Informed consent

Written informed consent for publication of the case details was obtained from the patient and the patient’s son.

### Disclosure statement

The authors have no conflicts of interest to declare.

### Additional remarks

The content of this article is an updated version of a case previously presented at the 46th Annual Meeting of the Japanese Society for Vascular Surgery (May 2018, Yamagata, Japan). This report includes additional mid- to long-term follow-up data not available at the time of the initial presentation.

### Author contributions

Operation: HS, SS, BK, GU, MF, and TY

Manuscript drafting: HS, SS, BK, YH, NO, SM, and TY

Study conception: HS, BK, and SS

Data collection: HS, BK, and SS

Analysis: HS

Investigation: HS and YH

Manuscript preparation: HS

Critical reviews and revision: all authors

Final approval of the article: all authors

Accountability for all aspects of the work: all authors.
